# Development and evaluation of a computerized algorithm for the interpretation of pulmonary function tests

**DOI:** 10.1371/journal.pone.0297519

**Published:** 2024-01-29

**Authors:** Yuh-Chin T. Huang, Luke Henriquez, Hengji Chen, Craig Henriquez

**Affiliations:** 1 Department of Medicine, Duke University Medical Center, Durham, NC, United States of America; 2 Department of Cognitive Science, Case Western University, Cleveland, OH, United States of America; 3 Department of Biomedical Engineering, Pratt School of Engineering, Duke University, Durham, NC, United States of America; SMS Medical College and Hospital, INDIA

## Abstract

Pulmonary function tests (PFTs) are usually interpreted by clinicians using rule-based strategies and pattern recognition. The interpretation, however, has variabilities due to patient and interpreter errors. Most PFTs have recognizable patterns that can be categorized into specific physiological defects. In this study, we developed a computerized algorithm using the python package (pdfplumber) and validated against clinicians’ interpretation. We downloaded PFT reports in the electronic medical record system that were in PDF format. We digitized the flow volume loop (FVL) and extracted numeric values from the reports. The algorithm used FEV1/FVC<0.7 for obstruction, TLC<80%pred for restriction and <80% or >120%pred for abnormal DLCO. The algorithm also used a small airway disease index (SADI) to quantify late expiratory flattening of the FVL to assess small airway dysfunction. We devised keywords for the python Natural Language Processing (NLP) package (spaCy) to identify obstruction, restriction, abnormal DLCO and small airway dysfunction in the reports. The algorithm was compared to clinicians’ interpretation in 6,889 PFTs done between March 1^st^, 2018, and September 30^th^, 2020. The agreement rates (Cohen’s kappa) for obstruction, restriction and abnormal DLCO were 94.4% (0.868), 99.0% (0.979) and 87.9% (0.750) respectively. In 4,711 PFTs with FEV1/FVC≥0.7, the algorithm identified 190 tests with SADI < lower limit of normal (LLN), suggesting small airway dysfunction. Of these, the clinicians (67.9%) also flagged 129 tests. When SADI was ≥ LLN, no clinician’s reports indicated small airway dysfunction. Our results showed the computerized algorithm agreed with clinicians’ interpretation in approximately 90% of the tests and provided a sensitive objective measure for assessing small airway dysfunction. The algorithm can improve efficiency and consistency and decrease human errors in PFT interpretation. The computerized algorithm works directly on PFT reports in PDF format and can be adapted to incorporate a different interpretation strategy and platform.

## Introduction

Pulmonary function test (PFT) is a clinical tool to evaluate the function of the respiratory system [1]. A complete test commonly includes spirometry, lung volumes and carbon monoxide diffusion capacity (DLCO). Clinicians then determine whether the tests have physiological abnormalities and, if so, catergorize the abnormalities into obstruction, restriction and gas transfer (DLCO) defects by comparing to reference values. The severity of the defect is expressed as a percent predicted of a reference value or the magnitude of statistical deviation from the mean predicted value (i.e., z-scores). International guidelines have been published to standardize the techniques for performing PFT [1–3] and the approach to interpret PFT [4].

Despite the existence of interpretation rules, previous studies have revealed significant inter-rater variability in assigning the correct diagnostic category and grading severity [5–7]. For instance, a recent study showed that pulmonologists’ identification of a restrictive pattern had a positive predictive value of 59% and a sensitivity of 75%, compared to normal or obstructive patterns [8]. This variability can also be attributed to poor test quality, which can result from patients having difficulty with the maneuvers, or cognitive errors made by the interpreters. These errors can stem from physical fatigue or mental lapses that occur when interpreters read many PFTs.

Another cause for the inter-rater variability is the assessment of small airway dysfunction. One commonly used method is to assess the flattening of the expiratory limb of FVL. This is currently evaluated by visual inspection of FVL by the PFT interpreters and is subject to significant inter-rater variabilities [9]. We have developed a small airway disease index (SADI) based on the angle formed by two linear segments: one is from peak expiratory flow to the point on the curve corresponding to 75% of the expiratory vital capacity, which roughly corresponds to the start of small airways [10–12], and the other is from this point to the end of the curve where the expiratory portion of the FVL intersects the x-axis [13].

Since a great majority of physiological abnormalities in PFTs have patterns that can be readily recognized, a computerized interpretation algorithm theoretically can be developed to perform initial reading, much like that in EKG [14,15]. In this study, we developed a computerized algorithm that can interpret basic physiological defects and small airway dysfunction. The interpretation was compared to clinicians’ interpretation in the reports.

## Materials and methods

All PFTs in this study were performed between March 1^st^, 2018, and September 30^th^, 2020. All PFTs were performed according to the guidelines set by the American Thoracic Society (ATS) for quality and repeatability. Only those tests that met the criteria were retained and reported. PFT reports were downloaded from the Duke electronic medical record system (EPIC) that were in PDF format on 10/22/2020. We digitized and extracted data points of the FVLs using a custom tool written in Python (version 3.7) (https://peps.python.org/pep-0537/). The data were entered into Excel and stored in Protected Analytics Computing Environment (PACE). The study was approved by the Duke Institutional Review Board (Pro00105365). The authors had access to information that could identify individual participants during or after data collection.

### Development of interpretation algorithm

**Fig 1:**
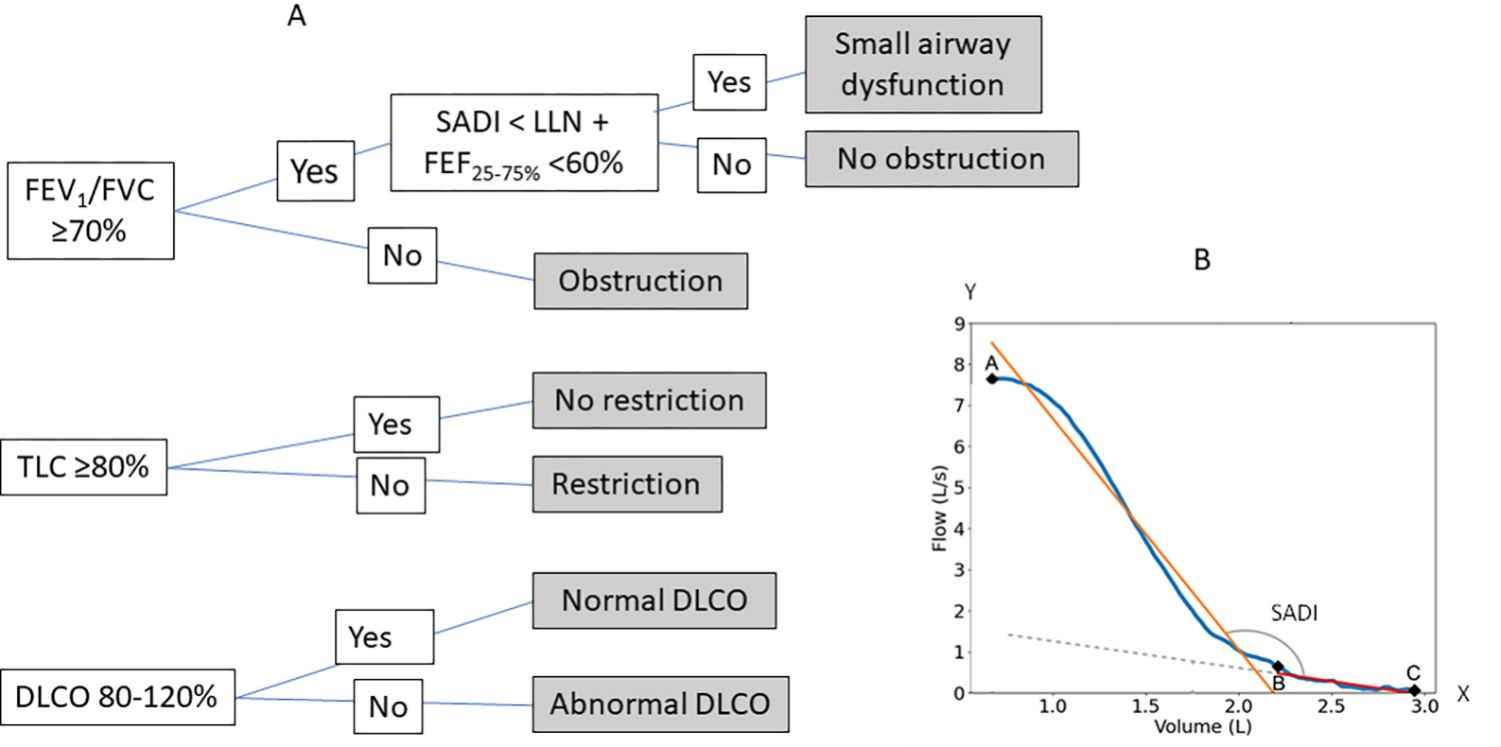
(A) Algorithm used to interpret PFTs. (B) Small airway disease index (SADI), which is the angle formed by two linear regression lines on the flow-volume loop (FVL). The first line is the fitting to the segment between the point at the peak expiratory flow (point A) and the point at 75% of the expiratory vital capacity (Point B). The second line is the fitting to the segment between point B and the point where the expiratory portion of the FVL intersects with the x-axis (point C)(Front Physiol 13:914972, 2022). LLN: Low limit of normal.

We used Python to develop an algorithm that follows the clinical guidelines for interpreting PFTs at Duke. We extracted PFT values using the Python package pdfplumber. The texts of clinician interpretation in the reports were recognized with the Python Natural Language Processing (NLP) package spaCy. For PFTs that had discordant interpretations between the algorithm and the clinician reports, we also manually checked to be sure the text recognition was accurate. The criteria for determining obstruction, restriction and abnormal DLCO are shown in Fig 1A. The algorithm checked FEV1/FVC and if FEV1/FVC was smaller than 0.7, the algorithm would label obstruction. For restriction, the algorithm checked TLC and if TLC was smaller than 80%pred, the algorithm would label restriction. For DLCO, the algorithm would consider DLCO <80%pred or >120%pred to be abnormal (reduced or elevated).

### Clinician interpretation of PFTs

These are the same criteria used by the clinician interpreters in our institution. The interpretation guidelines in our institution are based on those recommended by ATS/ERS [4]. The texts for obstruction, restriction and abnormal DLCO were embedded in specific macros in SentrySuite® (Vyaire Medical, Inc., Mettawa, IL). The clinicians simply need to key in the macros to display the text in the report without manual typing.

### Assessment of small airway dysfunction

The algorithm also included a small airway disease index (SADI) reported previously by our group [13]. The SADI is a measurement of the late expiratory flattening of the flow-volume loop (FVL). Briefly, after digitizing the expiratory portion of the FVL, we located the points A, B and C on the curve as shown in Fig 1B. Point A is the highest point of the FVL (peak expiratory flow). Point B is the point on the curve corresponding to 75% of the expiratory vital capacity, which corresponds to the start of small airways [10–12]. Point C is the end of the curve where the expiratory limb of the FVL intersects the x-axis. The SADI is the angle between the linear fitting of AB and BC.

### Statistical analysis

Data are expressed as mean ± standard deviation (SD). To determine the agreement between the interpretation by the algorithm and by clinicians, we calculated the agreement rate (agreement/(agreement+disagreement) and Cohen’s kappa [16]. All statistical analyses were performed using Microsoft Excel Office 16. A p<0.05 was considered statistically significant.

## Results and discussion

A total of 6,889 PFT reports were included. The agreement rates for obstruction, restriction and abnormal DLCO were 94.4% (Cohen’s kappa = 0.868), 99.0% (Cohen’s kappa = 0.979) and 87.9% (Cohen’s kappa = 0.750) respectively. There were 389 PFTs with discrepant interpretations for obstruction, 29 for restriction and 542 for DLCO.

**Fig 2:**
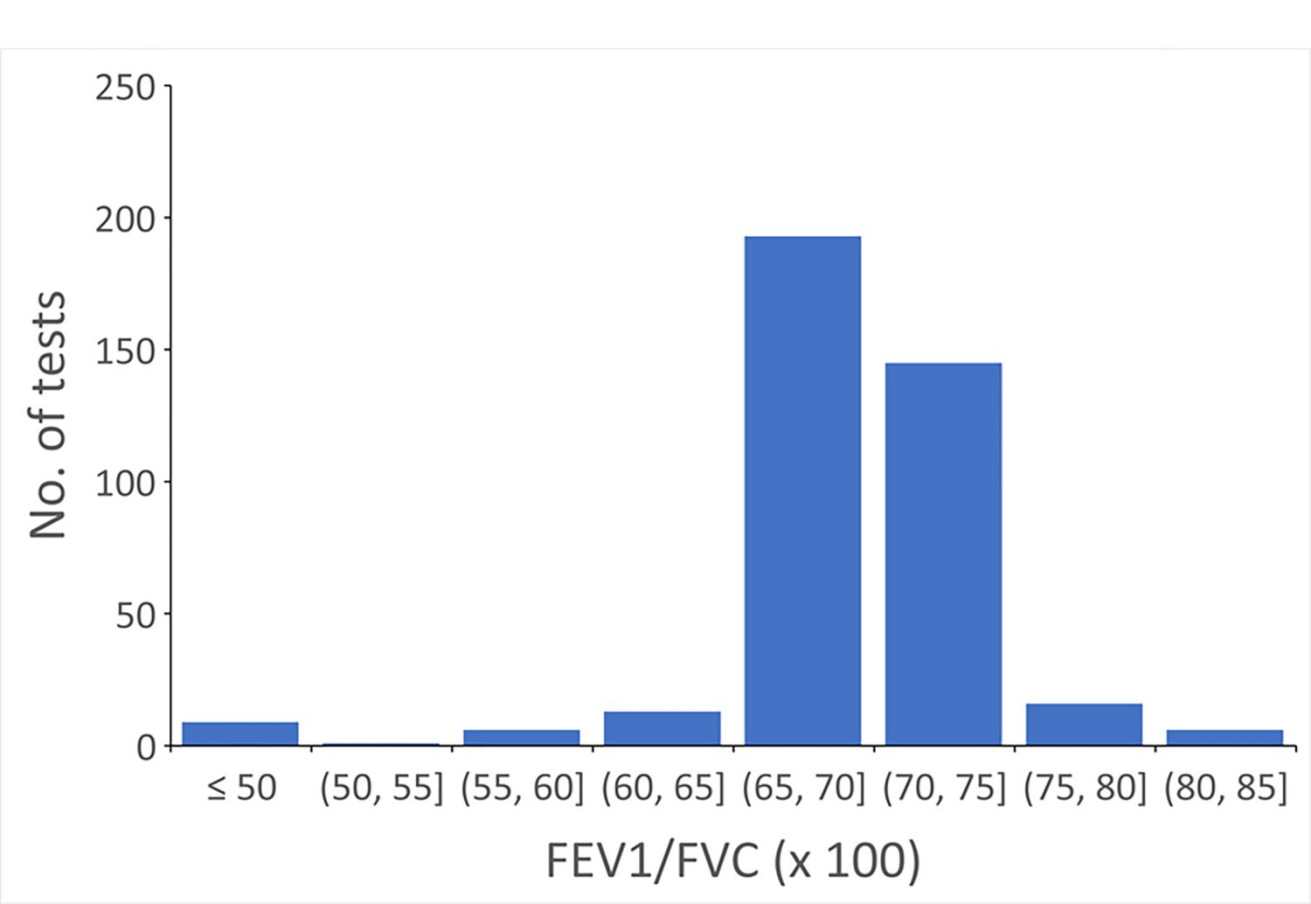
Distribution of FEV1/FVC in pulmonary function tests with discrepant interpretations between the algorithm and the clinicians.

The distribution of FEV1/FVC for the 389 PFTs with discrepant interpretations for obstruction is shown in Fig 2. Mean FEV1/FVC was 0.69±0.06 (median [interquartile range] = 0.70 [0.68,0.71]). There were 351 tests (90.2%) with FEV1/FVC between 0.65 and 0.75. There were 168 tests in which the algorithm indicated no obstruction, but the clinicians interpreted them as obstruction (43.2%). There were 221 tests in which algorithm indicated obstruction, but the clinicians interpreted them as no obstruction (56.8%).

**Fig 3:**
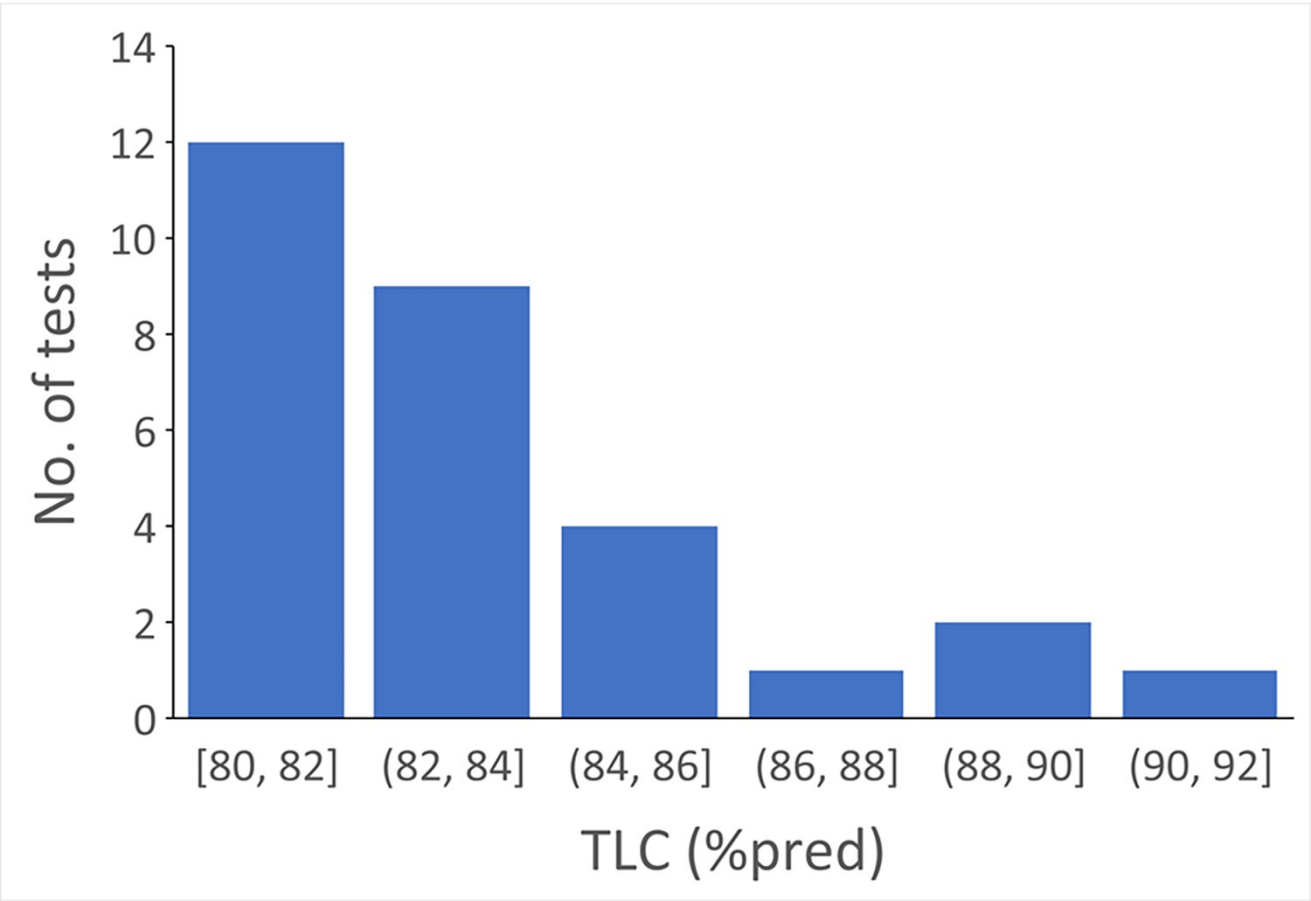
Distribution of total lung capacity (TLC) in pulmonary function tests with discrepant interpretations between the algorithm and the clinicians.

The distribution of TLC (%pred) for the 29 PFTs with discrepant interpretations for restriction is shown in Fig 3. The mean TLC was 83.3±2.8%pred (median [interquartile range] = 82.5 [81.2,85.0]). All 29 tests had TLC ≥ 80%pred and were interpreted by the algorithm as no restriction, but as restriction by the clinicians.

**Fig 4:**
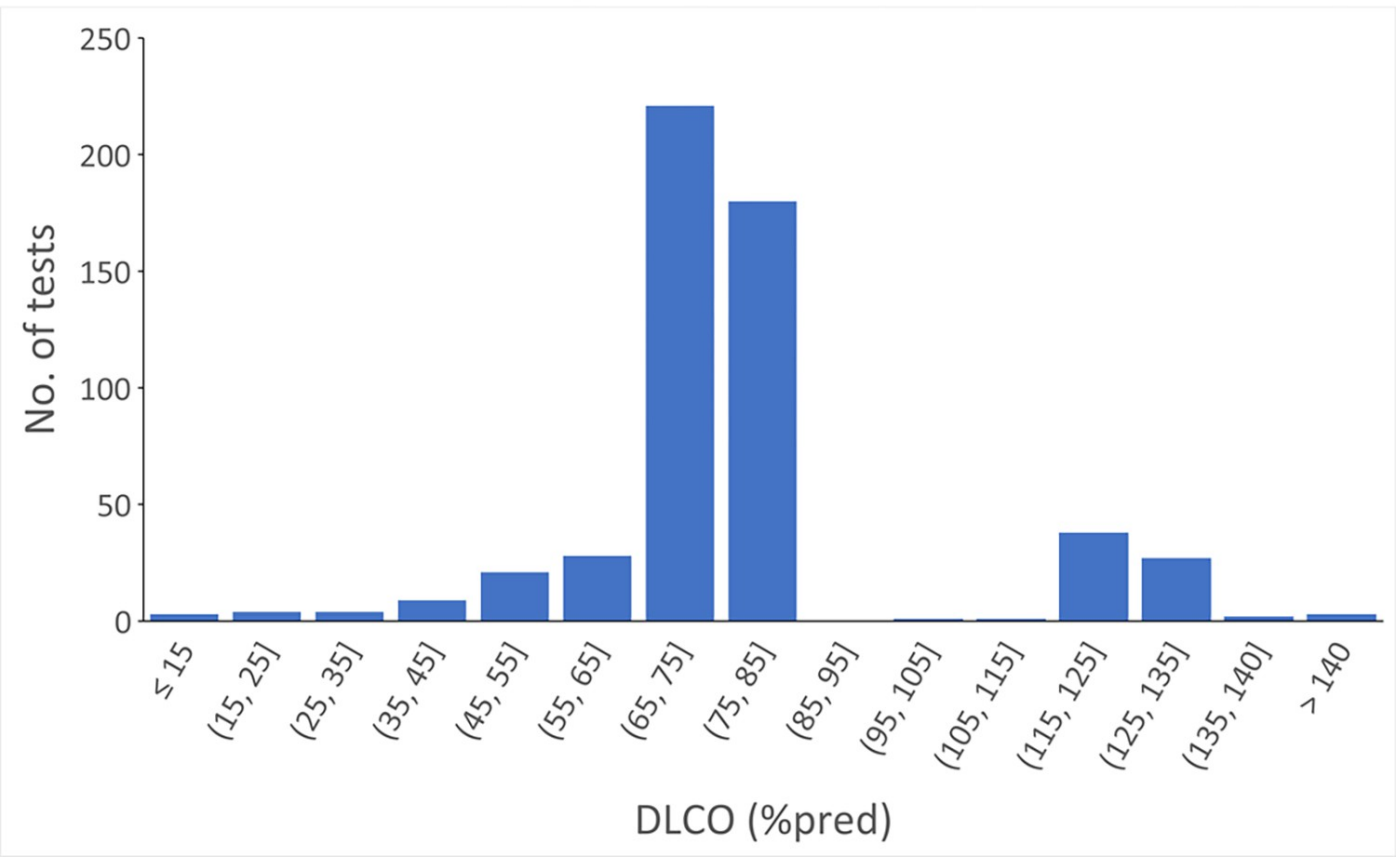
Distribution of DLCO in pulmonary function tests with discrepant interpretations between the algorithm and the clinicians.

The distribution of DLCO for the 542 PFTs with discrepant interpretations for abnormal DLCO is shown in Fig 4. The distribution of the discrepant tests had two modes. One mode consisted of DLCO greater than 120%pred. No clinicians interpreted these tests as abnormal (or elevated DLCO). The other mode consisted of DLCO mostly less than 80%pred. Only one test was interpreted by the algorithm as normal, but as abnormal by the clinicians. The remainder of the tests were interpreted as abnormal by the algorithm, but as normal by the clinicians.

**Fig 5:**
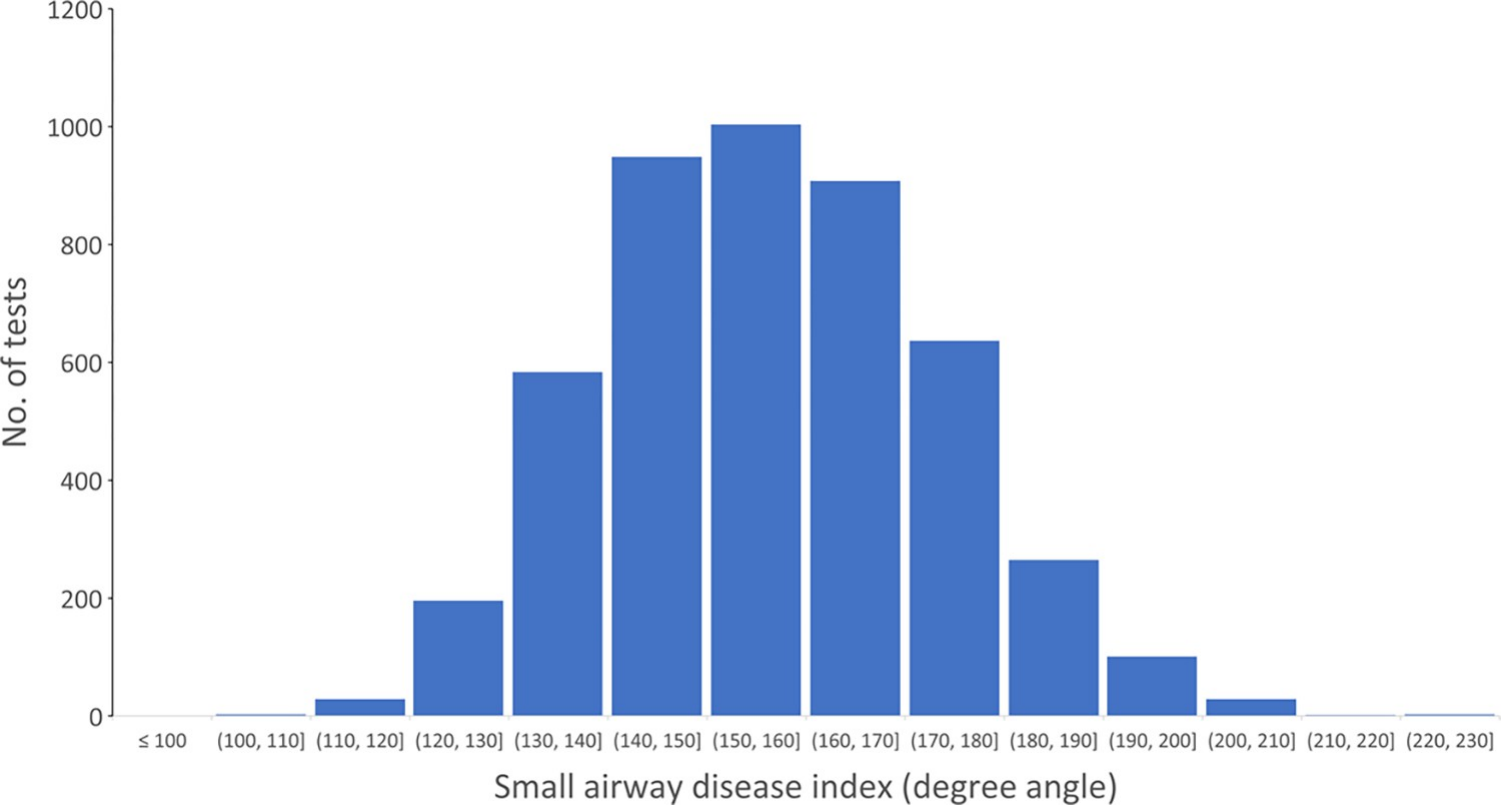
Distribution of small airway disease index (SADI in degree angle) in pulmonary function tests with FEV1/FVC ≥ 0.7.

In the 4,711 PFTs with FEV1/FVC≥0.7, we calculated SADI [13]. The distribution of SADI was Gaussian (Fig 5). The mean was 156.5 with an SD of 16.9. The median was 155.8. The LLN (1.64xSD) for SADI was 128.8. Using this LLN, the algorithm identified 190 tests with small airway dysfunction. Of these, clinicians also flagged 129 tests (67.9%) to have small airway dysfunction in the report. When SADI was greater than LLN, no clinicians indicated small airway dysfunction.

We further analyzed potential cognitive errors for tests with discrepant interpretation. For obstruction, the causes included missing clinician’s interpretation (n = 23), and no interpretation due to patient difficulty with the maneuver (n = 14) or lack of reference equation due to extremely advanced age (n = 1). For restriction, all 29 PFTs had normal TLC but decreased VC, a pattern that could be consistent with early restriction. For DLCO, the causes included missing the DLCO component of the interpretation (n = 32), no interpretation due to patient difficulty with the maneuver (n = 15) or inadequate inspiratory capacity that was judged by the clinicians to cause inaccurate results (n = 14). One test had DLCO of 43%pred but was interpreted as normal in the clinician’s report. Our results showed the computerized interpretation algorithm performed very well in identifying obstruction, restriction and abnormal DLCO compared to clinicians. The agreement rates were 88–99%. The majority of discrepancies were observed in tests that were in the “gray” zone, i.e., the tests with values near the normal cutoff or range. For these tests, clinicians might have used a different normal range or considered other factors, such as the shape of the FVL (for obstruction), race and ethnicity, and smoking status (when available) for interpretation.

Out of 389 spirometries with discrepant interpretation for obstruction, 351 (~90%) had FEV1/FVC between 0.65 and 0.75. This range is where the interpretation of airway obstruction is subject to significant variability. Clinicians likely judge the presence of airway obstruction using additional criteria, such as the concavity of the expiratory limb of the FVL, other PFT parameters (e.g., FEF25-75, RV), or patient’s information (e.g., body weight, smoking status). One study developed a concavity measure for FVL in adults and showed it tended to be abnormal in smokers who had normal numeric values in spirometry [17]. Another study quantified the concavity by measuring the rectangular area ratio, i.e., the ratio of the integrated area under the FVL between the two anchoring points, maximal expiratory flow and the zero flow and the entire area of the rectangle [18]. This measurement correlated with dynamic hyperinflation and exercise limitation in COPD patients. However, neither evaluated the discriminant power of the degree of concavity for the presence of airway obstruction.

The agreement rate for restriction was the highest. All discrepancies came from the PFTs that had normal TLC but decreased VC, a pattern that could be consistent with early restriction. This could easily be incorporated into our algorithm in the future.

The agreement rate for abnormal DLCO was also high, but lower than those for obstruction and restriction. A main reason for the discrepancy in DLCO interpretation may be a wider normal range (e.g., 70%-130% pred) some clinicians used during their interpretation. If the algorithm had used this wider normal range, the agreement rate would have increased to 97.8%. The algorithm also did not adjust for hemoglobin.

One unique feature of our computerized algorithm was the SADI that measured the flattening of the late expiratory limb of the FVL, an assessment of small airway dysfunction [13]. The assessment traditionally relied on visual inspection of the flattening that is subject to significant intra- and inter-rater variability. The SADI now provides a numeric value to assist the interpreters. Of the tests with SADI less than LLN, clinicians only flagged two-third of them. When SADI was greater than LLN, no clinicians called small airway dysfunction, indicating SADI has a high sensitivity. The clinical significance for SADI needs further investigation in the future. Our previous study that used a greater SADI showed that approximately 50% of the patients with small SADI had small airway disease on CT scans [13].

The algorithm also found other causes for the disagreement between the algorithm and the clinicians. For example, 32 DLCO and 25 spirometries were without interpretation. In one test, the clinician failed to identify DLCO of 43%pred as abnormal. These were likely inadvertent human errors from physical fatigue and mental lapses. Missing interpretation would have resulted in loss of revenue.

One limitation for the algorithm was the inability to determine the test quality. Some clinicians tended not to interpret the tests when they considered the performance of the tests to be suboptimal due to patient difficulty with the maneuvers, although there were no objective standards to determine the test quality. Multiple studies have used machine learning to assess the quality of spirometry [19–21]. These quality measures can be used to improve our algorithm in the future. Another limitation was the algorithm interpreted DLCO without taking into account the hemoglobin. This could decrease the agreement rate between the algorithm and the clinicians. Also our algorithm simply identified obstruction, restriction and abnormal DLCO. We will expand the pilot algorithm to include identification of air trapping, bronchodilator response, and the severity of the defects in future versions.

In conclusion, we developed a pilot computerized algorithm for PFT interpretation that performed well compared to clinicians. It had an added feature of SADI that can assist the clinicians in calling small airway dysfunction when visually assessing the FVL. The algorithm can decrease human errors and the burden for the clinicians and allows them to focus on a smaller proportion of the tests that need their attention. Our computerized algorithm can work directly on any PFT reports in PDF format. The codes can be altered to accommodate the different PFT report format in different institutions and vendors. It can also be adapted to incorporate different interpretation strategies, e.g., the LLN method, and add features such as severity and test quality.

## Supporting information

S1 Dataset (XLSX)

S1 File (ZIP)
